# Amyloid-Like Fibril Elongation Follows Michaelis-Menten Kinetics

**DOI:** 10.1371/journal.pone.0068684

**Published:** 2013-07-10

**Authors:** Katazyna Milto, Akvile Botyriute, Vytautas Smirnovas

**Affiliations:** Department of Biothermodynamics and Drug Design, Vilnius University Institute of Biotechnology, Vilnius, Lithuania; University of Maryland School of Medicine, United States of America

## Abstract

A number of proteins can aggregate into amyloid-like fibrils. It was noted that fibril elongation has similarities to an enzymatic reaction, where monomers or oligomers would play a role of substrate and nuclei/fibrils would play a role of enzyme. The question is how similar these processes really are. We obtained experimental data on insulin amyloid-like fibril elongation at the conditions where other processes which may impact kinetics of fibril formation are minor and fitted it using Michaelis-Menten equation. The correlation of the fit is very good and repeatable. It speaks in favour of enzyme-like model of fibril elongation. In addition, obtained 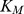 and 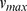 values at different conditions may help in better understanding influence of environmental factors on the process of fibril elongation.

## Introduction

First amyloid-like deposits were found more than a hundred years ago. Nowadays we know tens of different diseases which are related to protein amyloid-like fibril formation. Numbers of other, disease-unrelated proteins can aggregate into amyloid-like fibrils under certain conditions. One of the hypotheses proposes fibrillar structure to be generic for all proteins [1]. It is suggested that fibrils are formed via nucleated growth (also known as “nucleation-elongation”) mechanism. Nucleation is rather a stochastic process - even at identical conditions, lag times of the fibrillation may differ [2], [3]. Once the first nucleus is formed, elongation process starts. While just several molecules may be enough to form nucleus, it can recruit thousands of molecules upon fibril elongation. Elongation is usually faster process than nucleation. Moreover, fibrils can elongate under conditions, not favorable for nucleation. Taken together, it suggests that the main driving force of amyloid formation lays in elongation.

Several different approaches were used to describe the process of protein amyloid-like fibril formation as a mathematical function. From simple logistic equation to describe nucleated growth [4], [5] and first-order kinetics to describe elongation [4], [6], [7], to more complicated three-step polymerization model [8] and even comprehensive models taking into account a sum of possible events such as primary nucleation, elongation and secondary nucleation (as breaking or branching of fibrils) [9]–[11]. However, these models still have some issues. Most common is an imperfect fit when applied to an experimental data. It may be a problem of both, the model, which may miss some of the processes involved in the fibrillation, and the data, which may be affected by fibrillation-unrelated processes. Thus to get a perfect fit by any model, the process of aggregation must be strictly controlled to make sure the data would not involve any processes, not described by the model. Our idea was to design the most simple amyloid-like fibril formation experiment which would involve the least number of different processes and thus could be fitted by a simple model. The only aggregation process, which can be separated from others, is fibril elongation. It can be initiated by adding fibrils to a protein solution. Concentration of fibrils and conditions of the reaction can be optimized to avoid primary nucleation. Secondary nucleation can be minimized by avoiding any kind of agitation.

It was noticed that elongation of fibrils is very similar to an enzymatic reaction [12] and even analyzed using Michaelis-Menten equation [13]. Growing ends of the fibril play role of the enzyme active site while monomers or oligomers play role of the substrate. Increase in mass of fibril mimics the product of enzymatic reaction. We applied the best-known model of enzyme kinetics, Michaelis-Menten equation, to describe the process of amyloid-like fibril elongation. Typical enzymatic reaction involves substrate binding and catalysis steps and can be summarized as:
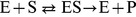
(1)where E is enzyme, S - substrate, ES - enzyme-substrate complex, and P - product. Elongation of fibrils can be described similar way:
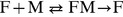
(2)where F is fibril, M - monomer (in fact, we can’t rule out the possibility of fibril elongation through attaching oligomers, but to keep the model as simple as possible we use M), and FM is a short-living complex, which exists from the moment when monomer attaches to the fibril until it’s completely incorporated. The rate of fibril elongation can be described by Michaelis-Menten equation:
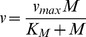
(3)where 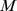 is concentration of monomer, 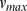 is the maximum rate (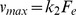, 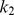 - rate constant of the FM complex maturation into longer fibrils, and 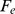 is concentration of fibril ends), and 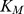 is Michaelis constant (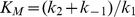, 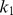 and 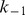 are rate constants of monomer binding to the fibril and FM complex dissociation, respectively).

## Results and Discussion

**Figure 1:**
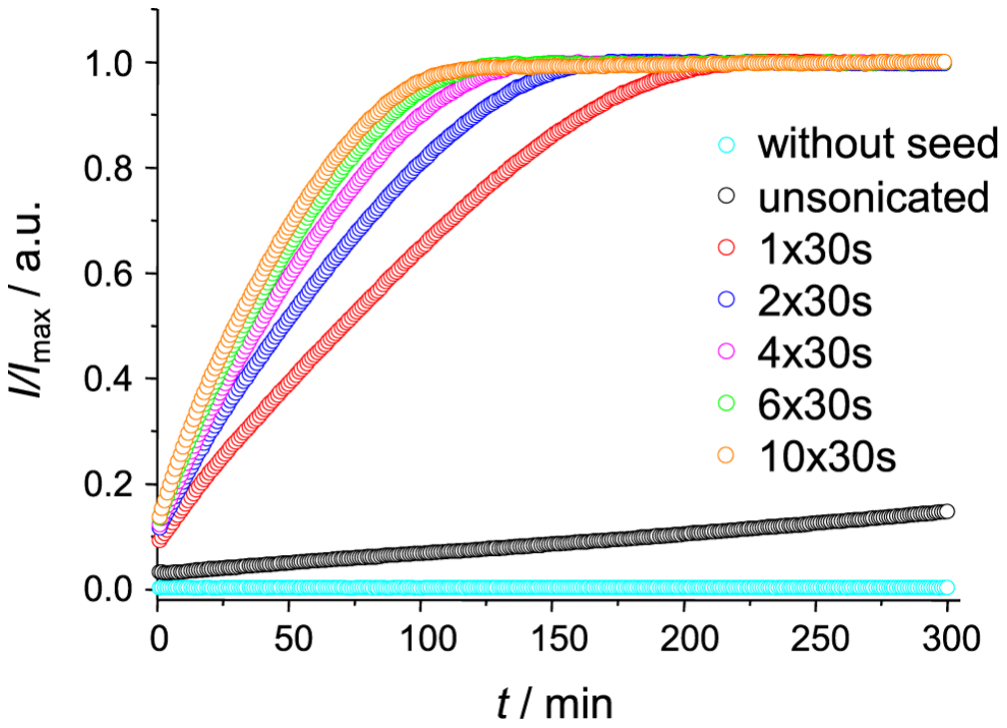
Influence of sonication. Different number of 30 s pulses were applied to fibrils before using them as seeds. Kinetics of elongation followed by Thioflavin T (ThT) fluorescence assay.

Ultrasonic treatment is often used for preparation of fibril seeds in studies of amyloid-like aggregation. It breaks fibrils into shorter pieces [14], [15] increasing number of fibril ends and accelerating elongation rate without a change of total protein concentration. In addition it also leads to more homogenous suspension [15], which is very important for reproducibility of results when working with high concentration of proteins. Scientists around the world use a number of different devices, such as different ultrasonic baths [15], [16] or homogenizers with microprobes [14], [17]. In each case there are a number of different factors which can influence efficiency of sonication. Due to this fact we checked the efficiency of our sonication setup before proceeding to further experiments. Sonication in a bath is much less efficient and highly dependent on properties of sample tubes. We observed difference in efficiency of sonication when using thin-walled tubes versus usual tubes (data not shown). Microprobes can be inserted into the sample, thus sonication efficiency is much less dependent on properties of tubes. Figure 1 shows a comparison of fibril elongation kinetics using seeds prepared by different sonication times. There is >10 times difference in rates of elongation while using unsonicated vs. sonicated fibrils as seeds. The time of sonication is also important, though every additional pulse of 30 seconds is less efficient, probably because shorter fibrils are mechanically more stable. For all further experiments we used ten 30 s cycles of sonication.

**Figure 2:**
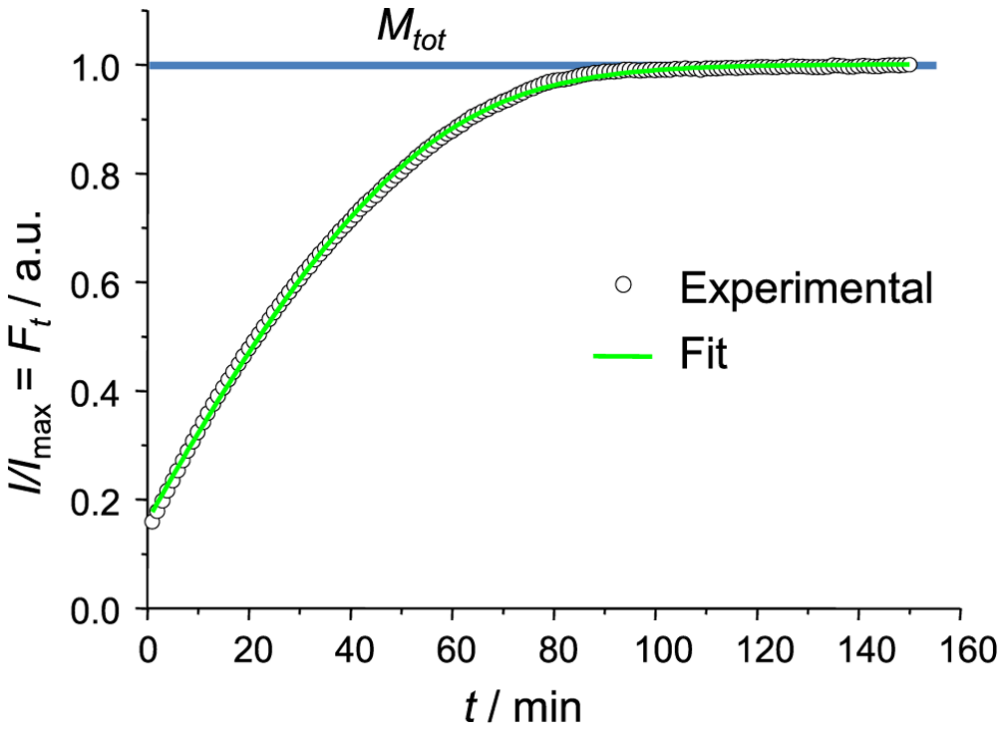
Insulin fibril elongation kinetics - experimental data and fitted curve. The curve reaches the plateau when all insulin is converted to fibrils (*F_t_*  =  *M_tot_*).

**Figure 3:**
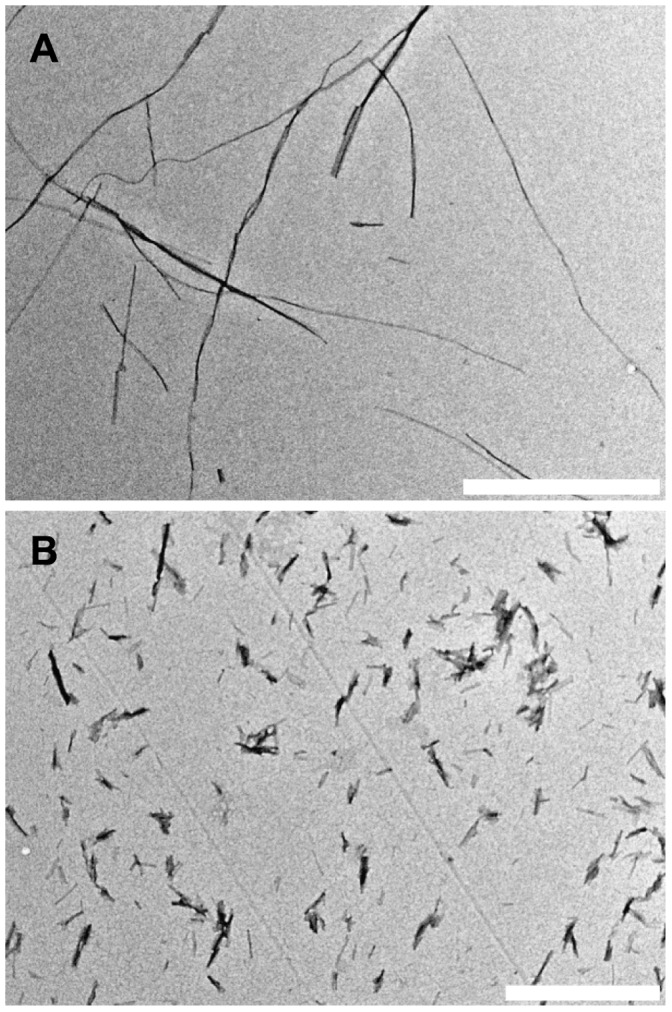
Transmission electron microscopy images. Spontaneously formed insulin amyloid-like fibrils before (A) and after (B) ten 30 s pulses of sonication (bar 1 *µ*m).

As seen in Figure 2, elongation curve, observed using 10% of sonicated seeds, shows a very good fit with Michaelis-Menten equation (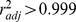). To ensure reliability of the fit, we tested errors of fitted 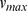 and 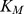 values for a number of samples within the same batch preparation, and for several different batches. In all repeats fitting errors were lower than 5%. Distribution of 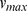 and 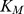 values within repeated experiments of the same batch preparation was very even, not exceeding fitting errors. The data looks much more scattered when different batches are compared (Table 1). Fluctuations of 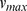 should be attributed to uneven number of fibril ends within each batch. It happens mostly due to the stochastic nature of spontaneous fibril formation. Although sonication helps to homogenize the sample, fibril lengths still may slightly differ from sample to sample. After sonication short pieces of fibrils tend to get together spontaneously (Figure 3), which is an additional factor for the differences between batches. It is clear that random events in preparation of seeds are way more important for the repeatability of 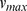 values than possible events during measurement of elongation kinetics and fitting errors. It is more problematic to explain differences in 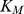 values. There is no direct correlation in fluctuations of 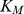 and 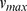 values between batches, which means no major differences in FM complex maturation rate. Thus the only valid guess would be shift of the equilibrium of FM complex formation. But such event also hardly explainable. It means the main responsibility for uncertain 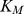 values goes to the measurement of kinetics and to the fitting. As theoretically Michaelis constant should be independent on batch preparation, we tried to increase precision of obtained 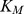 values by doing a global fit of all measurements with shared 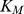. Correlation of the global fit was similar to independent fits (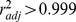). Obtained 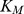 and 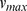 values fits average 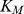 and 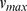 values obtained through independent fits (Table 1). It seems global fit does not significantly change the meaning of the data, but give more fine tuning of fitted values leading to better precision.

**Table 1:** Fitted *K_M_* and *v_max_* values.

	without NaCl		with 100 mM NaCl	
batch^a^	*K_M_* [*µ*m]	*v_max_* [*µ*m/min]	*K_M_* [*µ*m]	*v_max_* [*µ*m/min]
1	331.84±4.87^c^	22.30±0.23	98.45±4.68	20.23±0.35
2	260.29±13.47	11.70±0.22	59.26±1.65	13.17±0.19
3	249.77±7.31	15.94±0.24	58.86±4.47	14.90±0.14
4	438.26±22.12	20.79±0.66	185.95±8.73	20.60±0.48
5	246.22±6.09	15.60±0.18	72.55±2.17	16.33±0.30
6	289.68±5.77	22.48±0.36	131.01±7.38	25.45±1.01
7	279.88±8.79	17.49±0.26	78.56±7.01	20.49±0.74
8	316.10±4.88	20.35±0.18	98.74±3.73	21.49±0.27
9	276.95±9.58	15.00±0.23	71.36±2.56	16.81±0.15
average	298.80±45.80	17.96±2.86	94.97±31.59	18.83±2.93
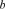 global fit	268.28±4.50	17.26±2.06	79.77±3.05	17.074±1.77

a Preparation and measurement of batches 1–5 and 6–9 were done by different persons.

b fit with shared *K_M_*.

c standard errors for all data were calculated using Student’s t-distribution at P = 0.05.

**Figure 4:**
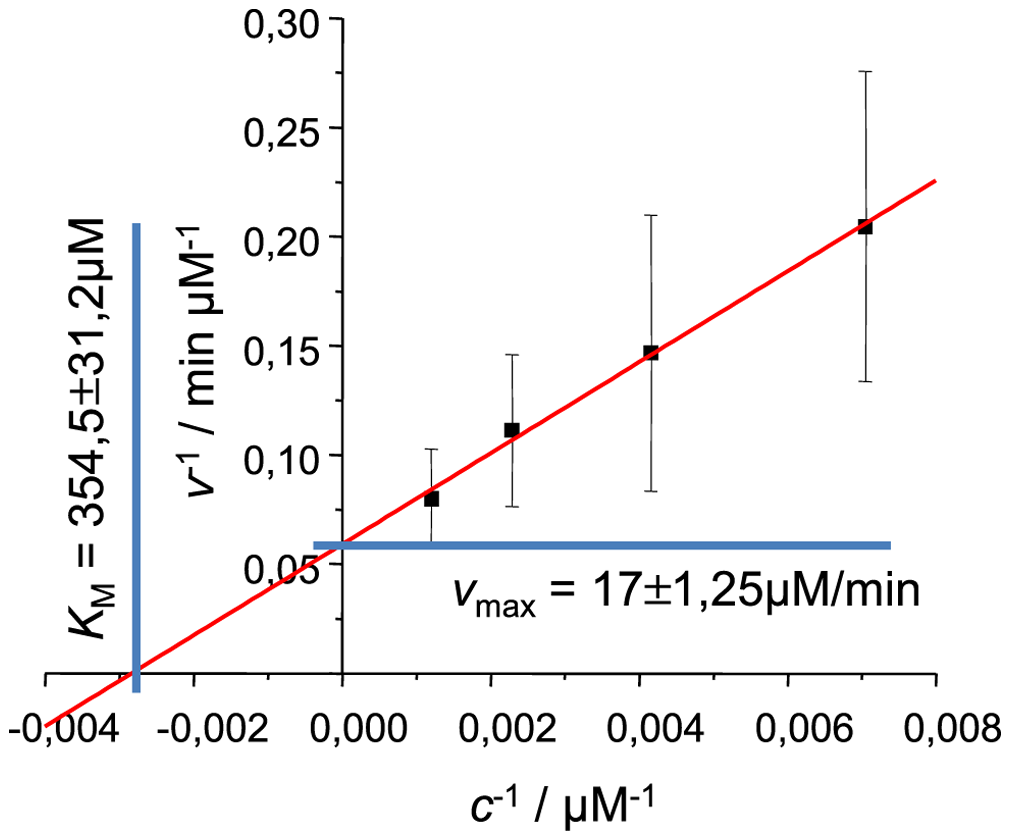
Lineweaver-Burk plot for fibril elongation. Rates of elongation were measured at the point 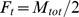, errors of elongation rates were estimated using the data from 5 different batches of seeds.

For an additional test, we added the same amount of seeds to different concentrations of insulin solution and used Lineweaver-Burk plot as a traditional approach to determine 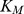 and 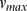. As seen in figure 4, 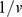 dependence on 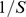 is linear and determined 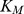 and 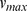 are comparable to the values, obtained by curve fitting.

**Figure 5:**
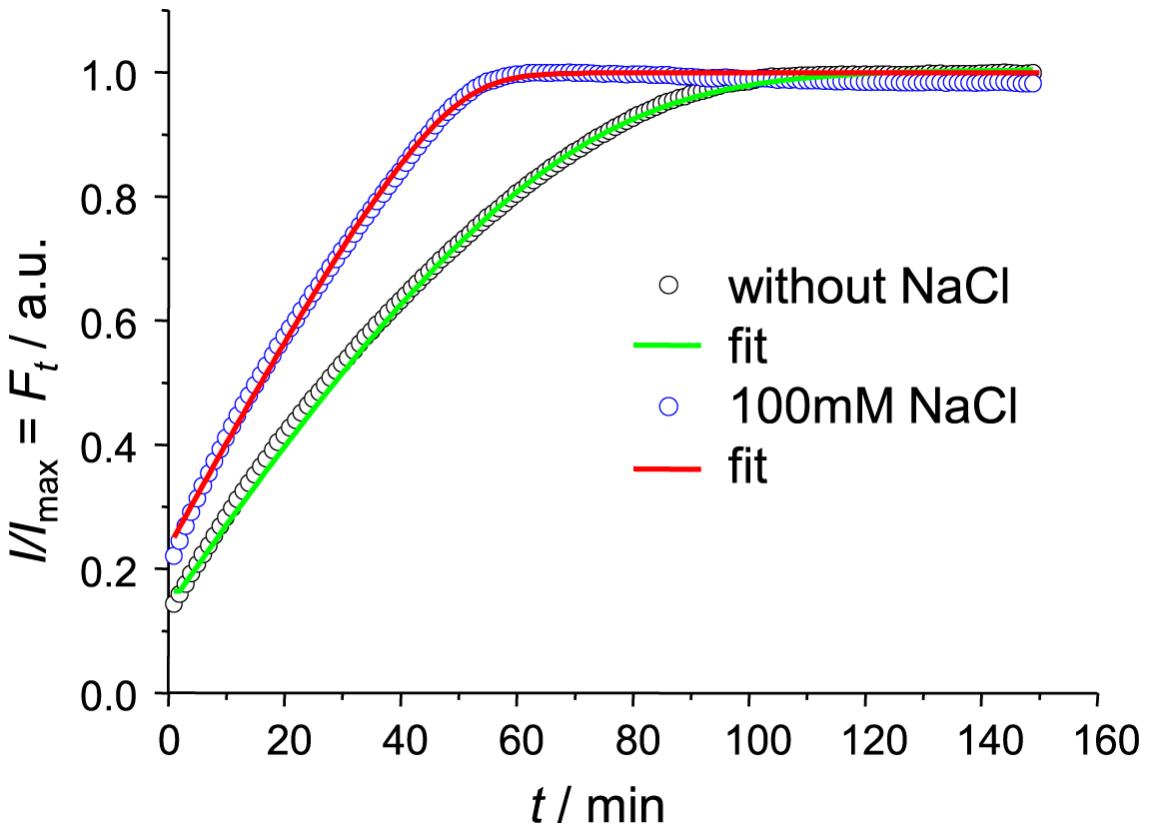
Comparison of insulin fibril elongation kinetics in absence and presence of 100 mM NaCl.

As it is well known, the presence of NaCl may affect kinetics [5] and even mechanism [18] of insulin amyloid-like fibril formation. To get data for an additional test of enzyme-like elongation model we did parallel seeding experiments in the presence of 100 mM NaCl using the same batch seed preparations. Data curves were a bit worse quality when compared to the ones without salt due to a slow drift of data points after reaching plateau (Figure 5). Nevertheless it had only minor effect on the quality of the fit (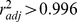). Fitting data revealed no major differences in 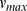 values between samples with and without NaCl. It means rate of FM complex maturation into longer fibril is not affected by ionic strength. 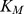 values in the presence of NaCl is 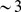 times lower than in the absence of salt. It means shift of the equilibrium towards FM complex formation. It is an expectable event: at low pH insulin molecules are positively charged so fibril-monomer electrostatic interactions are not favourable, while increase of ionic strength of the environment lowers impact of charge and increase the rate of fibril-monomer association.

We demonstrated that Michaelis-Menten enzyme-like kinetics can be a very good model to describe fibril elongation when experimental conditions minimize events of primary and secondary nucleation. By comparing a number of batches we showed possible errors when relying on the single experiment and an increase of precision when all repeats are used together. Finally, analysis of experimental fibril elongation data using Michaelis-Menten equation can better explain impact of environmental conditions. The advantage of such approach is ability to study elongation almost independently from other events, such as nucleation, termination or fragmentation. Any of these events affects the number of fibril ends, which leads to substrate-concentration-independent changes of elongation rate. In such case 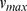 becomes variable, which means that a good fit using Michaelis-Menten equation is very unlikely. From the other side, the same reason makes the application of this approach rather narrow. Nevertheless, in light of possible generic nature of amyloid-like fibrils [1] and an emerging idea of prion-like nature of amyloid fibrils [19]–[23] a simple model with clearly defined variables has a chance to evolve into a tool for classification of amyloid-like fibrils by their elongation potential at different conditions (which at some point may evolve to “infectivity potential”).

## Materials and Methods

Recombinant human insulin was purchased from Sigma Aldrich (91077C). Insulin amyloid-like fibrils were prepared by incubation of fresh 5 mg/ml insulin solution (in 100 mM phosphate buffer (PB), pH2; prepared in secure lock tubes, Fisher Scientific UK, (FB74071)) at 60°C for 24 hours with 300 rpm agitation (using MHR 23 thermomixer, Ditabis, Germany). To homogenize aggregated material and maximize concentration of fibril ends, fibrils were subjected to ultrasonic treatment. 1 ml of fibrils were put into 2 ml tube and homogenized for 10 minutes using Bandelin Sonopuls 3100 ultrasonic homogenizer equipped with MS73 tip (using 50% of power, cycles of 30 s/30 s sonication/rest, total energy applied to the sample per cycle ∼ 0.56 kJ). The sample was kept on ice during the sonication. Right after the treatment, 1 part of the fibrils were mixed with 9 parts of the fresh 5 mg/ml (to obtain Lineweaver-Burk plot 10, 2.5, and 1.25 mg/ml were also used) insulin solution (100 mM PB, pH2, with or without 100 mM NaCl), containing 50 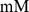 Thioflavin T (ThT). Each batch was divided into 20 ml aliquots (in 200 ml thin-wall PCR tubes). Elongation kinetics was measured at constant 37°C temperature using Corbett Rotor-Gene 6000 real-time analyzer. Increase of ThT fluorescence intensity upon fibril formation was observed using green channel (excitation 470 nm; emission 510 nm). Data was collected for up to 900 minutes, taking reads every minute.

ThT fluorescence curves were normalized by dividing each point by the maximum intensity of the curve. Assuming ThT fluorescence curves represent change of fibril mass in time, the fitting equation looks like:
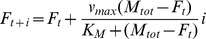
(4)where 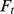 is fibrillated protein concentration at time 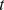, 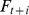 - fibrillated protein concentration at time 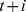, 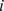 - iteration time interval, and 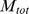 is the total protein concentration. There are 4 constants (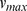, 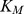, 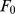, and 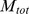), which can be fitted using least square method. Figure 2 shows a fit of our experimental data using 1 minute iteration interval (same as our data spacing). Direct curve fit gives values of the constants in arbitrary units. As 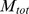 used in our experiments is known (5 mg/ml), other constants can be converted from arbitrary units to real units using the same proportion. Fitting was performed using Origin 8 software.

For electron microscopy 3 *µ*l of 20 times diluted (with water) samples were applied on formvar/carbon coated 300 mesh copper grids (Agar scientific) for 1 minute. Staining was performed by applying 3 *µ*l of 2% uranyl acetate aqueous solution for 30 seconds. Images were acquired using FEI Morgagni 268 microscope.

## References

[1] Chiti F, Dobson CM (2006) Protein misfolding, functional amyloid, and human disease. Annu Rev Biochem 75: 333–66. 16756495 10.1146/annurev.biochem.75.101304.123901

[2] Hortschansky P, Schroeckh V, Christopeit T, Zandomeneghi G, Fändrich M (2005) The aggregation kinetics of Alzheimer’s beta-amyloid peptide is controlled by stochastic nucleation. Protein Sci 14: 1753–9. 15937275 10.1110/ps.041266605 PMC2253354

[3] Foderà V, Librizzi F, Groenning M, van de Weert M, Leone M (2008) Secondary nucleation and accessible surface in insulin amyloid fibril formation. J Phys Chem B 112: 3853–8. 18311965 10.1021/jp710131u

[4] Naiki H, Gejyo F (1999) Kinetic analysis of amyloid fibril formation. Method Enzymol 309: 305–18. 10.1016/s0076-6879(99)09022-9 10507032

[5] Nielsen L, Khurana R, Coats A, Frokjaer S, Brange J, et al (2001) Effect of environmental factors on the kinetics of insulin fibril formation: elucidation of the molecular mechanism. Biochemistry 40: 6036–46. 11352739 10.1021/bi002555c

[6] Esler WP, Stimson ER, Ghilardi JR, Vinters HV, Lee JP, et al (1996) In vitro growth of Alzheimer’s disease beta-amyloid plaques displays first-order kinetics. Biochemistry 35: 749–57. 8547255 10.1021/bi951685w

[7] Naiki H, Hashimoto N, Suzuki S, Kimura H (1997) Establishment of a kinetic model of dialysisrelated amyloid fibril extension in vitro. Amyloid 232: 223–232.

[8] Cannon MJ, Williams AD, Wetzel R, Myszka DG (2004) Kinetic analysis of beta-amyloid fibril elongation. Anal Biochem 328: 67–75. 15081909 10.1016/j.ab.2004.01.014

[9] Xue WF, Homans SW, Radford SE (2008) Systematic analysis of nucleation-dependent polymerization reveals new insights into the mechanism of amyloid self-assembly. P Natl Acad Sci USA 105: 8926–31. 10.1073/pnas.0711664105 PMC2440360 18579777

[10] Knowles TPJ, Waudby Ca, Devlin GL, Cohen SIA, Aguzzi A, et al (2009) An analytical solution to the kinetics of breakable filament assembly. Science 326: 1533–7. 20007899 10.1126/science.1178250

[11] Cohen SIA, Vendruscolo M, Dobson CM, Knowles TPJ (2011) Nucleated polymerisation in the presence of pre-formed seed filaments. Int J Mol Sci 12: 5844–52. 22016630 10.3390/ijms12095844 PMC3189754

[12] Chatani E, Ohnishi R, Konuma T, Sakurai K, Naiki H, et al (2010) Pre-steady-state kinetic analysis of the elongation of amyloid fibrils of beta(2)-microglobulin with tryptophan mutagenesis. J Mol Biol 400: 1057–66. 20595042 10.1016/j.jmb.2010.05.071

[13] Scheibel T, Bloom J, Lindquist SL (2004) The elongation of yeast prion fibers involves separable steps of association and conversion. P Natl Acad Sci USA 101: 2287–92. 10.1073/pnas.0308754101 PMC356943 14983002

[14] Serio TR, Cashikar AG, Kowal AS, Sawicki GJ, Moslehi JJ, et al (2000) Nucleated conformational conversion and the replication of conformational information by a prion determinant. Science 289: 1317–21. 10958771 10.1126/science.289.5483.1317

[15] Chatani E, Lee YH, Yagi H, Yoshimura Y, Naiki H, et al (2009) Ultrasonication-dependent production and breakdown lead to minimum-sized amyloid fibrils. P Natl Acad Sci USA 106: 11119–24. 10.1073/pnas.0901422106 PMC2708754 19564620

[16] Dzwolak W, Smirnovas V, Jansen R, Winter R (2004) Insulin forms amyloid in a strain-dependent manner: an FT-IR spectroscopic study. Protein Sci 13: 1927–32. 15169954 10.1110/ps.03607204 PMC2279922

[17] Saborío GP, Permanne B, Spagnolo S (2001) Sensitive detection of pathological prion protein by cyclic amplification of protein misfolding. Nature 411: 810–3. 11459061 10.1038/35081095

[18] Smirnovas V, Winter R (2008) Revealing different aggregation pathways of amyloidogenic proteins by ultrasound velocimetry. Biophys J 94: 3241–6. 18192359 10.1529/biophysj.107.123133 PMC2275694

[19] Westermark GT, Westermark P (2010) Prion-like aggregates: infectious agents in human disease. Trends Mol Med 16: 501–7. 20870462 10.1016/j.molmed.2010.08.004

[20] Brundin P, Melki R, Kopito R (2010) Prion-like transmission of protein aggregates in neurodegenerative diseases. Nat Rev Mol Cell Bio 11: 301–7. 20308987 10.1038/nrm2873 PMC2892479

[21] Eisele YS, Obermüller U, Heilbronner G, Baumann F, Kaeser Sa, et al (2010) Peripherally Applied A{beta}-Containing Inoculates Induce Cerebral {beta}-Amyloidosis. Science 980: 10–13. 10.1126/science.1194516 PMC3233904 20966215

[22] Frost B, Diamond MI (2010) Prion-like mechanisms in neurodegenerative diseases. Nat Rev Neurosci 11: 155–9. 20029438 10.1038/nrn2786 PMC3648341

[23] Lee SJ, Desplats P, Sigurdson C, Tsigelny I, Masliah E (2010) Cell-to-cell transmission of non-prion protein aggregates. Nat Rev Neurol 6: 702–706. 21045796 10.1038/nrneurol.2010.145 PMC4996353

